# Keeping track of moving targets

**DOI:** 10.7554/eLife.109627

**Published:** 2025-12-02

**Authors:** Renata Batista-Brito, Geoffrey Terral

**Affiliations:** 1 Dominick P Purpura Department of Neuroscience, Albert Einstein College of Medicine Bronx United States; 2 Department of Psychiatry and Behavioral Sciences, Albert Einstein College of Medicine Bronx United States; 3 Department of Genetics, Albert Einstein College of Medicine Bronx United States

**Keywords:** cell tracking, calcium imaging, development, neocortex, neuronal activity, Mouse

## Abstract

A new method for tracking the activity of individual neurons day after day in the growing brain has revealed a key developmental transition in neuronal activity.

**Related research article** Majnik J, Mantez M, Zangila S, Bugeon S, Guignard L, Platel JC, Cossart R. 2025. Longitudinal tracking of neuronal activity from the same cells in the developing brain using Track2p. *eLife* **14**:RP107540. doi: 10.7554/eLife.107540.

The brain changes rapidly in the days after birth. Neurons grow, circuits reorganize, and patterns of neuronal activity shift as animals transition from early sensory experiences to active exploration. Although many researchers have studied these changes by comparing neuronal activity at isolated time points during development, tracking the same set of neurons as the brain matures has been technically challenging. Now, in eLife, Jure Majnik and colleagues report an elegant solution to this challenge that enables daily tracking of the same set of neurons in young mice during a period of rapid brain growth (Majnik et al., 2025).

Visual inspection and manual annotation can be used to track individual cells in sparse populations over periods of days. However, the automated tracking of dense populations of neurons during early development is difficult because the cortex expands, stretches, and remodels rapidly as the brain grows. Moreover, neurons change their appearance, some neurons die, and their relative positions drift as the tissue changes. This means that the methods used to track dense population of neurons in adult animals often fail when applied to young pups.

To tackle this problem, Majnik and colleagues at INMED, INSERM and Aix-Marseille University – including Jean-Claude Platel and Rosa Cossart as joint corresponding authors – used an established technique called two-photon calcium imaging to image the same set of neurons from the same cortical region in mouse pups every day from eight days after birth to fourteen days after birth. This period is a critical stage of developmental during which the brain undergoes dramatic anatomical and functional changes. Majnik et al. then used a new computational method called *Track2p* to align these images in a way that compensates for natural growth: instead of forcing the images to fit a fixed template, the method adjusts each day’s image relative to the previous one (Figure 1). This step smooths out the changes caused by tissue expansion.

**Figure 1.**
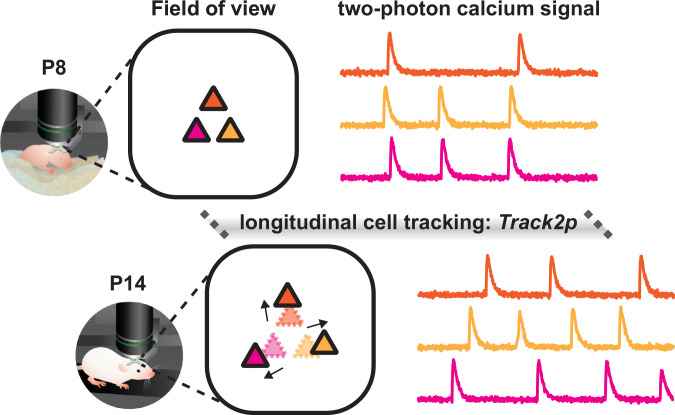
Longitudinal tracking of individual neurons in the developing brain. Schematic of three neurons (colored triangles) recorded with two-photon calcium imaging (colored traces on right) eight days after birth (P8; top), and fourteen days after birth (P14: bottom). The algorithm developed by Majnik et al., *Track2p*, can track individual neurons during this period despite the brain growing rapidly and neurons being displaced from their original positions (black arrows). Majnik et al. found that neuronal activity is more diverse and less synchronous at P14, compared to P8, with a key transition occurring around eleven days after birth (P11; not shown).

After the alignment step, the program compares the position and shapes of the neurons in the images and matches the neurons in the different images based on the amount of spatial overlap (with greater overlap indicating a higher likelihood that the shapes represent the same neuronal cell). Majnik et al. confirmed the accuracy of this approach through careful manual inspection, showing comparable performance between *Track2p* and human labelling.

Although the manual tracking of sparse, individual cells over multiple days is feasible, extending this to hundreds of neurons would require substantial effort and time. The method developed by Majnik et al. overcomes this limitation, making it possible to reliably track hundreds of neurons in each animal, and thus investigate the changes in neuronal activity that occur during development. The new approach helped Majnik et al. to discover a surprisingly abrupt shift in brain activity around eleven days after birth (P11). Before this time, neurons tend to be active together in large, highly synchronized bursts. After P11, their activity becomes more diverse and desynchronized. This transition also marks an increase in the complexity of activity patterns, suggesting that the circuit is gaining the capacity to encode more information.

The study also reveals how the developing cortex begins to respond to the animal’s own movements. By coupling the calcium recordings with video monitoring, Majnik et al. found that the movement of the pups had little influence on neuronal activity during the early stages of development. However, a clear relationship emerged after P11: some neurons were more active when the pups moved, while other neurons were less active during movement. This movement-related activity was stable and could be used to predict the animal’s behavior. Because the same neurons could be tracked throughout development, Majnik et al. were able to determine when these new patterns of activity emerged, and to demonstrate their stability across brain development. A number of developmental processes – including the maturation of inhibitory circuits, changes in neuromodulatory signals, and the beginning of active exploration – are likely to influence the timing of this transition.

The work of Majnik and colleagues shows that important developmental transitions can be missed when neuronal activity is examined only at isolated time points, rather than longitudinally in the same cells across a number of days. Beyond its biological findings, this work also makes available a method that has exceptional potential: *Track2p* is an open-source and user-friendly tool that will allow researchers to monitor neuronal development with unprecedented resolution.
